# Local recurrence of submucosal invasive colorectal cancer after endoscopic submucosal dissection revealed by copy number variation

**DOI:** 10.1002/deo2.208

**Published:** 2023-01-31

**Authors:** Yu Okazawa, Kiichi Sugimoto, Yuki Ii, Takahiro Irie, Megumi Kawaguchi, Aya Kobari, Hirotaka Momose, Yuki Tsuchiya, Kota Amemiya, Shunsuke Motegi, Ryoichi Tsukamoto, Kazumasa Kure, Kumpei Honjo, Hisashi Ro, Rina Takahashi, Shingo Kawano, Masaya Kawai, Shun Ishiyama, Makoto Takahashi, Yutaka Kojima, Yuichi Tomiki, Atsushi Arakawa, Takashi Yao, Kaishi Satomi, Yuko Matsushita, Koichi Ichimura, Kazuhiro Sakamoto

**Affiliations:** ^1^ Department of Coloproctological Surgery Juntendo University Faculty of Medicine Tokyo Japan; ^2^ Department of Human Pathology Juntendo University Faculty of Medicine Tokyo Japan; ^3^ Department of Pathology, Kyorin University School of Medicine Tokyo Japan; ^4^ Department of Brain Disease Translational Research Faculty of Medicine Juntendo University Tokyo Japan

**Keywords:** cecal cancer, copy number variation, endoscopic submucosal dissection, local recurrence, submucosal invasive colorectal cancer

## Abstract

We report a case in which analysis of copy number variation revealed local recurrence of submucosal invasive colorectal cancer after curative endoscopic submucosal dissection (ESD). An 86‐year‐old man with a history of abdominoperineal resection of the rectum for rectal cancer underwent resection with ESD for early‐stage sigmoid cancer 5 cm away from the stoma opening. At the same time, ileocecal resection was performed for advanced cecal cancer. Twelve months after ESD, advanced cancer occurred in the area of the ESD lesion. It was unclear if the cancer was a local recurrence after ESD, implantation of cecal cancer, or a new lesion. Copy number variation analysis performed for the three lesions revealed that the new lesion originated from residual tumor cells from ESD and was unlikely to be cecal cancer.

## INTRODUCTION

Endoscopic submucosal dissection (ESD) is a minimally invasive treatment that enables en‐bloc resection regardless of the size of a colorectal lesion and allows a detailed pathological examination while permitting functional preservation of organs.^1^ Local recurrence of submucosal invasive colorectal cancer after curative ESD is rare. Both tumor cell implantation and incomplete resection leaving a residual tumor are recognized as causes of local recurrence after ESD.^2^ Post‐ESD local recurrence due to implantation requires tumor cells to exfoliate into the intestinal lumen and implant and grow on the dissection surface created during ESD.^3^ In the case reported here, we performed sigmoidectomy for a lesion in an area where ESD had been performed. Local recurrence after ESD or implantation of advanced cecal cancer was suspected because the patient had cecal cancer at the time ESD was performed for early‐stage sigmoid cancer. Since an exact diagnosis was not possible using pathological findings alone, we investigated the three lesions based on copy number variation (CNV). The lesion that developed in the ESD area was concluded to be a local recurrence because the two lesions in the sigmoid colon had identical CNV features, while cecal cancer had different features. Thus, CNV analysis was useful in distinguishing local recurrence after ESD from implantation of advanced cecal cancer.

## CASE REPORT

The patient was an 86‐year‐old man who was found to have anemia in a blood test and underwent a colonoscopy. He had undergone abdominoperineal resection of the rectum for rectal cancer 28 years ago.

**FIGURE 1:**
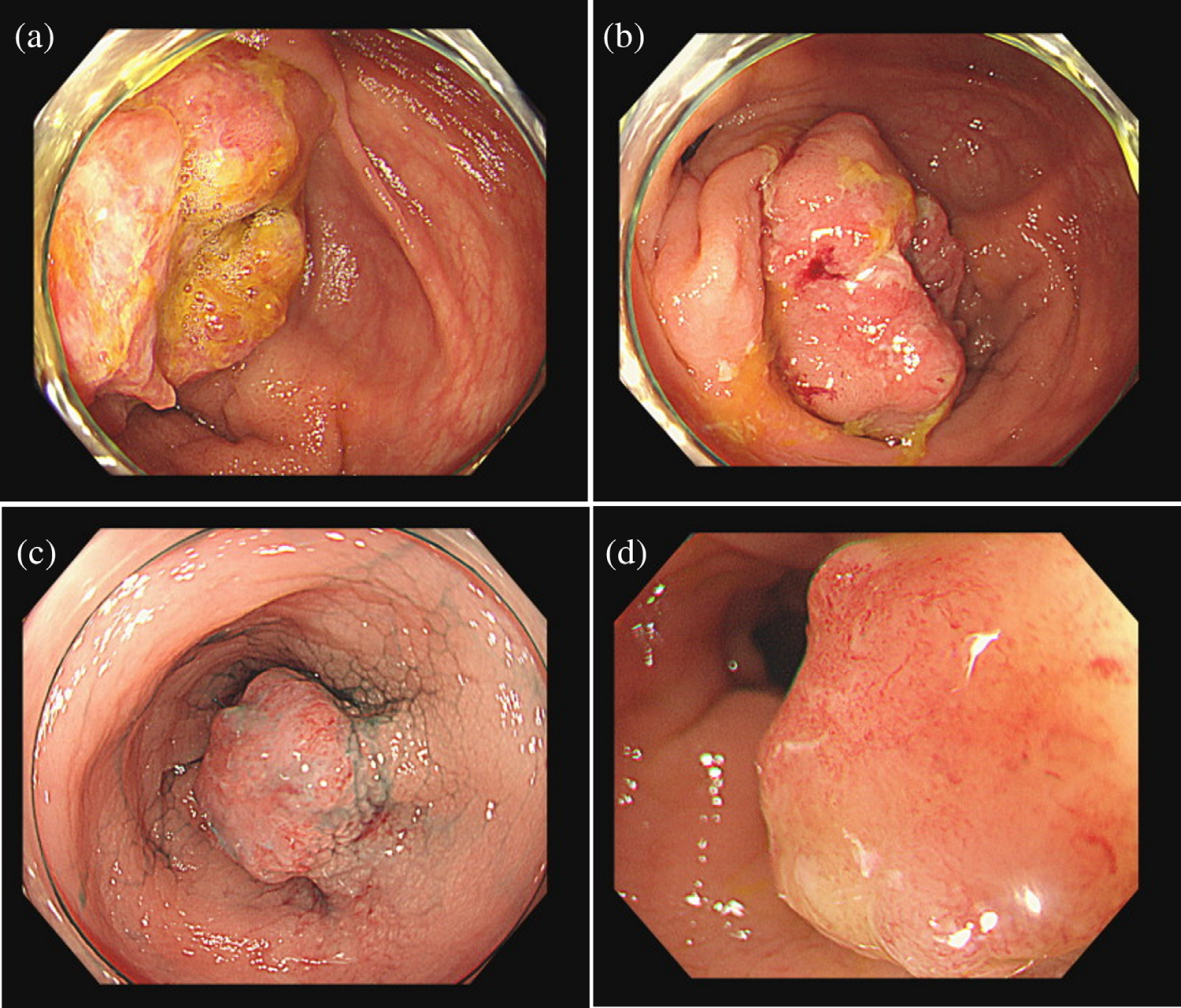
Colonoscopy revealed two tumors. (a, b) A 40‐mm type 2 tumor in the cecum. (c, d) A Is+IIc lesion in the sigmoid colon 5 cm from the stoma opening.

**FIGURE 2:**
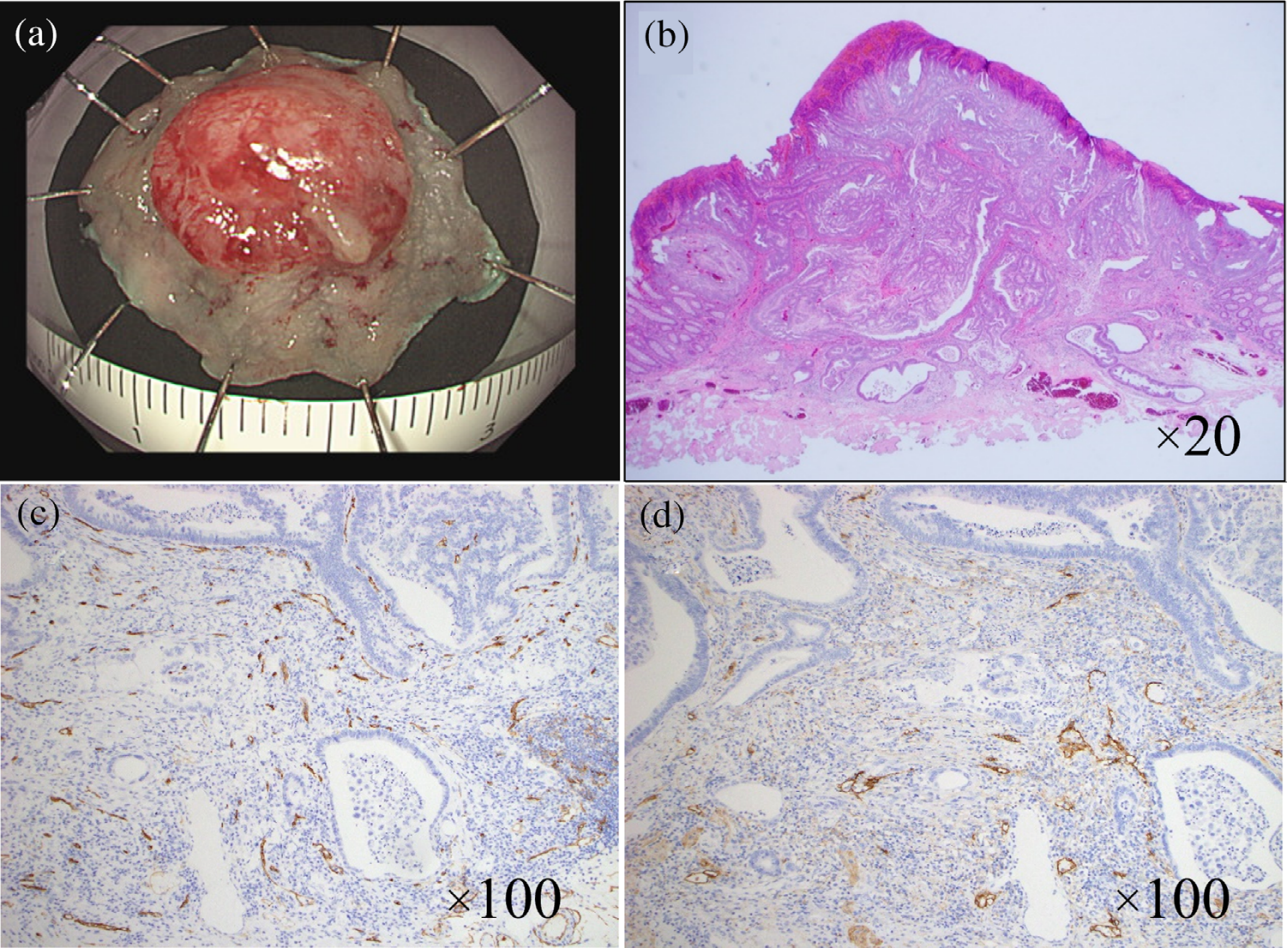
Endoscopic submucosal dissection procedure for the Is+IIc lesion in the sigmoid colon. (a) The tumor was resected en bloc. The resected specimen showed a 0‐I type tumor measuring 9×9 mm. The lateral margin was negative. Microscopic examination revealed a well‐differentiated adenocarcinoma and budding grade 1. The tumor had invaded the submucosa and infiltrated 2000 μm from the surface layer. Immunostaining revealed negative vascular invasion (b–d). (b) Hematoxylin and eosin staining. Magnification of ×20. (c) CD34 staining. Magnification of ×100. (d) D2‐40 staining. Magnification of ×100.

The colonoscopy revealed two tumors: a 40‐mm type 2 tumor in the cecum and a Is+IIc lesion in the sigmoid colon 5 cm away from the stoma opening (Figure 1). Computed tomography showed no distant metastasis. Consequently, the patient was diagnosed with synchronous multiple colorectal cancer, and ileocecal resection, sigmoid colon resection, and reconstruction of the stoma were judged to be necessary. However, due to the age of the patient, we decided to perform ESD on the Is+IIc lesion for a complete resection biopsy and to determine the appropriate surgical procedure based on pathological findings. It was very difficult to maintain the field of view because the lesion was located very close to the stoma opening, but the tumor was resected en bloc in 30 min (Figure 2a).

Grossly, the tumor measured 9×9 mm and the lateral margin was negative (Figure 2a). Microscopic examination revealed a well‐differentiated adenocarcinoma, a negative vascular invasion, a negative vertical margin, a negative horizontal margin, and budding grade 1^4^ (Figure 2b–d). The tumor had invaded the submucosa to a distance of 2000 μm from the surface layer. The distance from the deepest point of the lesion to the resection margin was 450 μm. The submucosally invasive colorectal cancer with only deep submucosal invasion as a risk factor had a low risk for recurrence. Considering the patient's advanced age and unwillingness, we decided not to perform surgery on this lesion. Therefore, only ileocecal resection with D3 lymph node resection was performed. Histopathological examination of a resected intestinal fragment revealed moderately differentiated adenocarcinoma, a 42×34 mm type 2 tumor invading the subserosa, and metastasis to six lymph nodes: tub2, T3/SS, N2a, Ly1c, V0, PM0, and DM0. Adjuvant chemotherapy was not given because the patient did not want this treatment.

**FIGURE 3:**
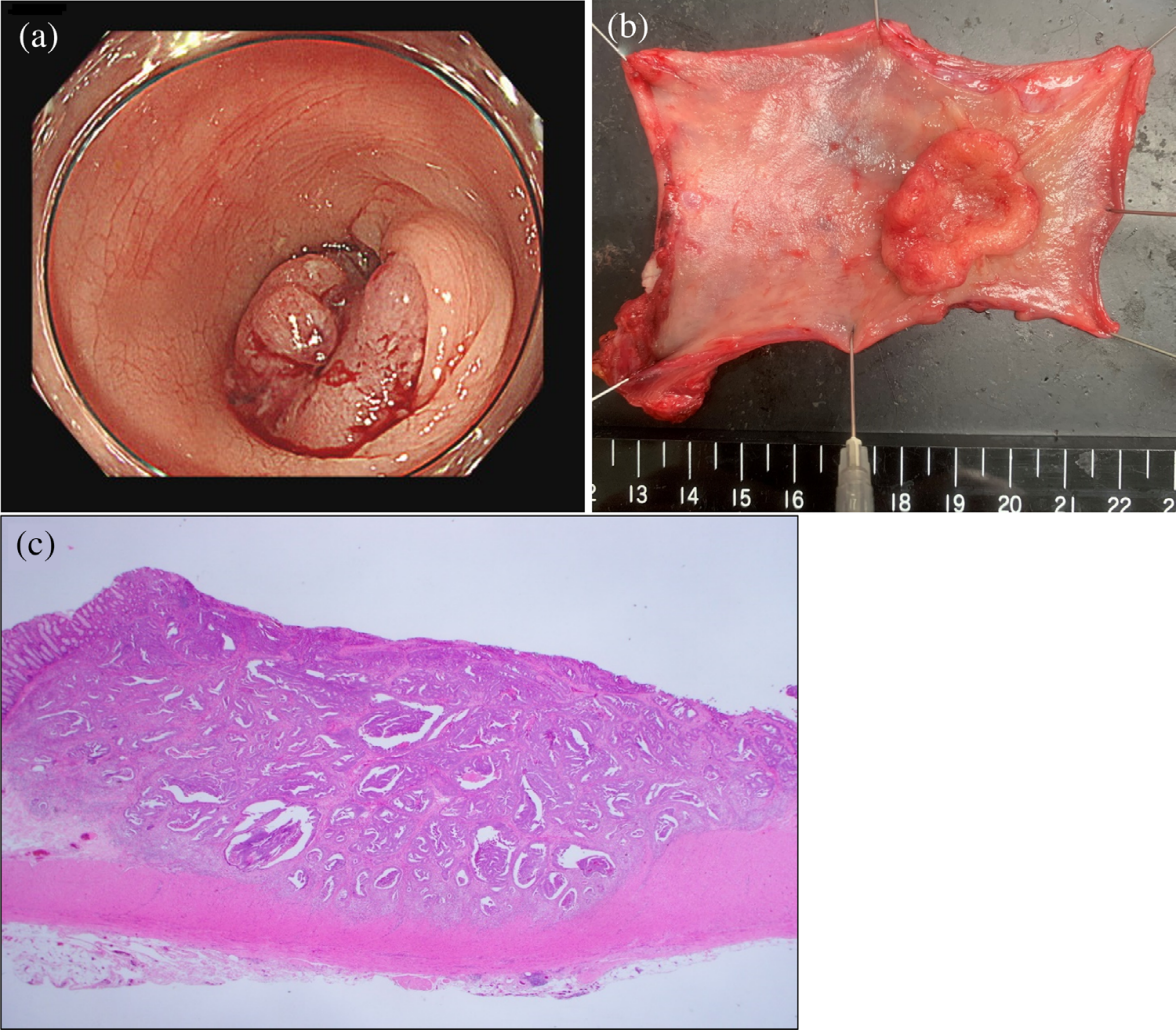
(a) Surveillance colonoscopy showed a type 2 tumor near the stoma opening. (b) A resected specimen from the operation showed a type 2 tumor measuring 35×30 mm. An endoscopic submucosal dissection scar was not detected. (c) Hematoxylin and eosin staining ×12.5. Histopathological examination revealed moderately differentiated adenocarcinoma, which was similar to the endoscopic submucosal dissection specimen.

Twelve months after ESD, the patient made an outpatient visit with a complaint of bleeding from the stoma. A surveillance colonoscopy showed a type 2 tumor near the stoma opening (Figure 3a). Post‐ESD scarring was not observed. A biopsy showed well‐differentiated adenocarcinoma, Group 5. Therefore, sigmoidectomy and stoma reconstruction were performed (Figure 3b). Histopathological examination revealed moderately differentiated adenocarcinoma that had invaded the muscular layer, with no lymph node metastases (Figure 3c). Microsatellite instability has not been examined.

It was not clear whether the tumor was a local recurrence after ESD, implantation of cecal cancer, or a new lesion. Therefore, CNV analysis was performed for the three lesions using a specimen from ESD, the recurrent tumor, and cecal cancer. The study was approved by the Institutional Review Board at Juntendo University Hospital (E21‐0353).

Genomic DNA was extracted as described previously.^5^ The tumor area most suitable for microdissection was recorded by directly marking the representative H&E stained section. Microdissection of cancer tissue from formalin‐fixed paraffin‐embedded sample was performed on three 10 μm unstained sections. The microdissected tissue sections were digested with GeneRead DNA FFPE Kit (QIAGEN, Germantown, MD, USA) and the resulting DNA (500 ng) was bisulfite converted with the EZ DNA Methylation Kit (Zymo Research, Irvine, CA, USA) and samples were processed according to the manufacturer's instructions. Raw IDAT files from the Infinium Methylation EPIC array (Illumina, San Diego, CA, USA) were processed using the minfi package (version 1.34.0) in R statistical environment (version 4.0.2), and quality control was performed.^5^ Mset objects generated from the raw IDAT files were used as the input data for CNV analysis using the conumee package (version 1.22.0). Using the genome annotations, more than 840,000 probes were used for further analysis. Copy number loci proceeded by conumee package were taken as the average of each chromosomal short arm (p) and long arm (q) using R. A widely used heuristic to identify gain or loss of each chromosome arm is determined to use a symmetrical absolute cut‐off of ±0.1 for conumee processed data. Automatic scoring was verified by manual assessment of the respective loci for each profile on a conumee plot. Probes assigned to chromosomes X and Y were excluded from the analysis.

**FIGURE 4:**
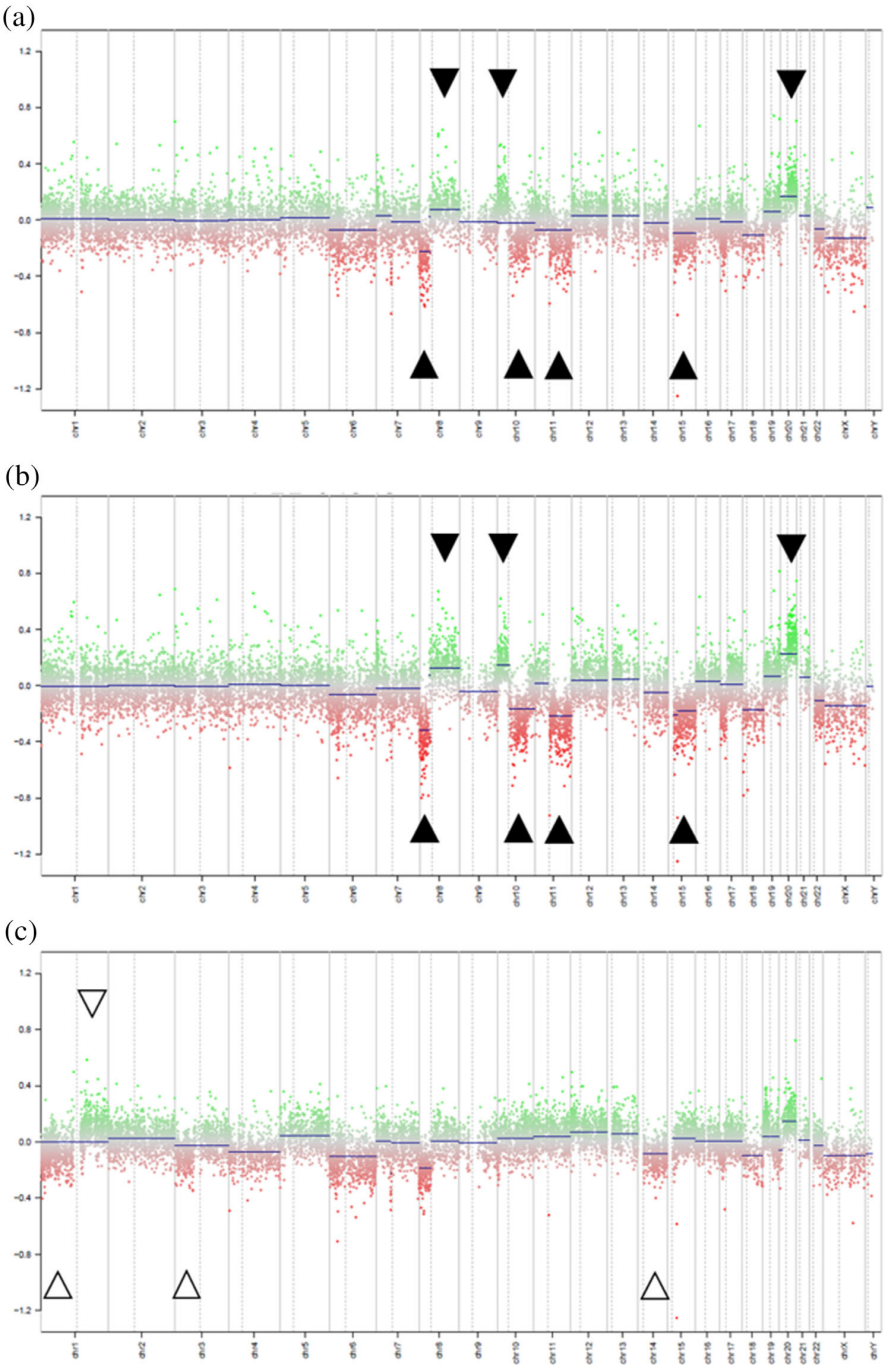
In conumee plots, analysis of the Infinium Methylation EPIC array showed that the copy number profiles for the endoscopic submucosal dissection lesion (a), and the recurrent tumor (b) were practically identical, sharing 8p loss, 8q gain, 10p gain, 10q loss, 11q loss, 15q loss, and 20q gain. In contrast, the cecal cancer (c) showed 1p loss, 1q gain, 3p loss, and 14q loss, which were not observed in A and B, while 10p gain, 10q loss, 11q loss, and 15q loss were absent.

Conumee plots showed that the copy number profiles in the ESD lesion and recurrent tumor were practically identical, sharing 8p loss, 8q gain, 10p gain, 10q loss, 11q loss, 15q loss, and 20q gain (Figure 4). In contrast, the cecal cancer showed 1p loss, 1q gain, 3p loss, and 14q loss, which were not observed in the other two samples, while 10p gain, 10q loss, 11q loss, and 15q loss were absent (Figure 4). These results suggest that the ESD lesion and the recurrent tumor were derived from cells of an identical origin, while the cecal cancer may have developed independently. It was thus deduced that the recurrence originated from cancer cells that remained after ESD.

## DISCUSSION

In this case, it was unclear if a newly detected lesion was a local recurrence after ESD or implantation of advanced cecal cancer. Pathological findings only permitted speculation with regard to the origin of the lesion in the sigmoid colon. Morgan found that carcinoma cells that exfoliated into the intestinal lumen did not survive on the normal mucosa, but did survive on a raw surface, such as that created by ESD.^6^ Inoue et al. suggested that intraluminal lavage of more than 1000 ml may be needed to remove exfoliated tumor cells after colorectal ESD.^7^ To our knowledge, the mechanism of local recurrence by implantation has only been reported for a rectal lesion, where exfoliated tumor cells are likely to accumulate. In our case, the ESD scar was located near the stoma opening and free viable tumor cells are unlikely to implant in this region.

Recurrence from a residual tumor can also arise from the horizontal margin and via lymphatic or venous invasion.^2^ Such invasion is sometimes detected in additional immunostaining or deeper sectioning of the lesion. In our case, immunostaining was performed, but deeper sectioning was not performed, so the possibility of recurrence through vascular invasion could not be denied completely. Emmanuel et al. detected microscopic residual adenoma after EMR in five of 21 cases (23.8%) at the base where submucosal tissue was additionally resected for a research purpose.^8^ This finding supports the hypothesis that microscopic residual adenoma is a potential mechanism of recurrence after endoscopic resection.^8^

The ability to evaluate clonal relatedness (i.e., whether clinically identified paired tumors, such as primary and locally recurrent tumors, share a common ancestor) is crucial in the development of a precise treatment strategy.^9^ A wide variety of analytical approaches have been assessed for the investigation of clonal relationships using bulk DNA.^9^ Waldman et al. showed the usefulness of somatic CNV to assess the clonal concordance of primary and recurrent ductal carcinoma in situ breast lesions.^10^ Our results suggest that the evaluation of CNV can also be used to determine the clonal concordance of primary and locally recurrent tumors in colorectal cancer. We believe that this study is the first to use CNV analysis to identify the origin of a locally recurrent tumor after curative ESD in colorectal cancer. This shows the value of CNV in revealing the clonal concordance of a locally recurrent tumor with a primary tumor after ESD. The local recurrence probably arose from a residual lesion at the base of the endoscopic resection.^8^ Thus surveillance colonoscopy is necessary after ESD, even if the horizontal margin, the lymphatic, and the venous invasion are negative.

## CONFLICT OF INTEREST

None.

## ETHICS STATEMENT

The study was conducted ethically in accordance with the World Medical Association Declaration of Helsinki.
